# Supplementary material for: Controlling spatiotemporal pattern formation in a concentration gradient with a synthetic toggle switch
Source: Mol Syst Biol. 2020 Jun 12;16(6):e9361. doi: 10.15252/msb.20199361 (PMC7290156; doi:10.15252/msb.20199361)
Supplement: Supplementary file 1 — Appendix [file MSB-16-e9361-s001.pdf]

Appendix

Controlling spatiotemporal pattern formation in a concentration gradient with a synthetic toggle switch

Içvara Barbier[1], Rubén Perez-Carrasco[2,3*] and Yolanda Schaerli[1*]

[1] Department of Fundamental Microbiology, University of Lausanne, Biophore Building, 1015 Lausanne, Switzerland

[2] Department of Mathematics, University College London, Gower Street, WC1E 6BT London, UK

[3] Department of Life Sciences, Imperial College London, South Kensington Campus, London, UK

*Correspondence: r.perez-carrasco@imperial.ac.uk and yolanda.schaerli@unil.ch

Table of contents

Appendix figure S1

Appendix figure S2

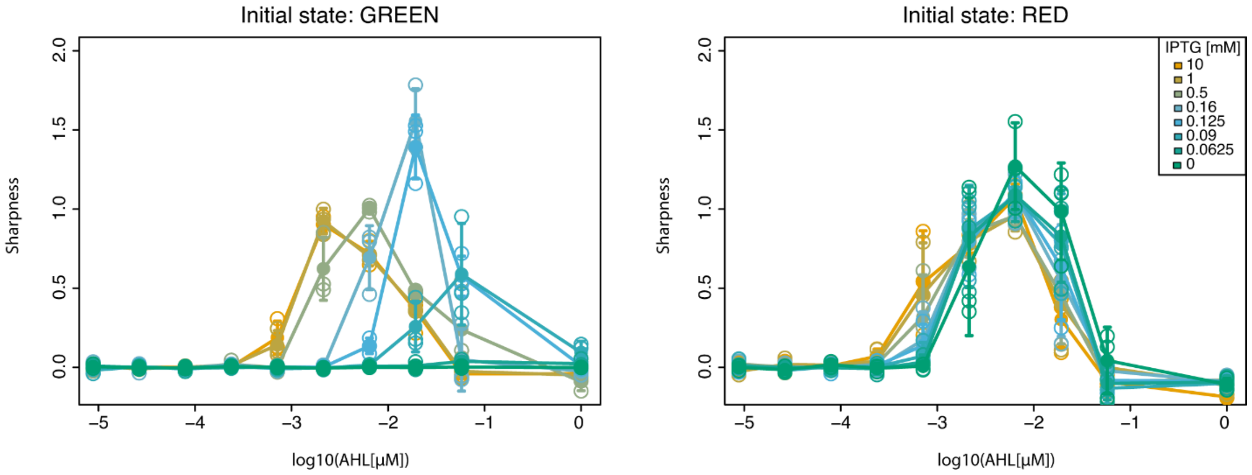

Appendix Figure S1: Effect of IPTG on the sharpness of the boundary

Quantification of the sharpness of the boundary across AHL gradient of data in figure 2E. The sharpness was calculated as the difference in red intensity between two AHL concentration divided by the difference of AHL concentration (log10), The equation was $(y_i\text{-}y_{i+1})/(\log10(x_i)\text{-}\log10(x_{i+1}))$. The higher and narrower a peak is, the sharper is the transition to the other state. Mean (full circles) and standard deviation of 3 biological replicates (empty circles).

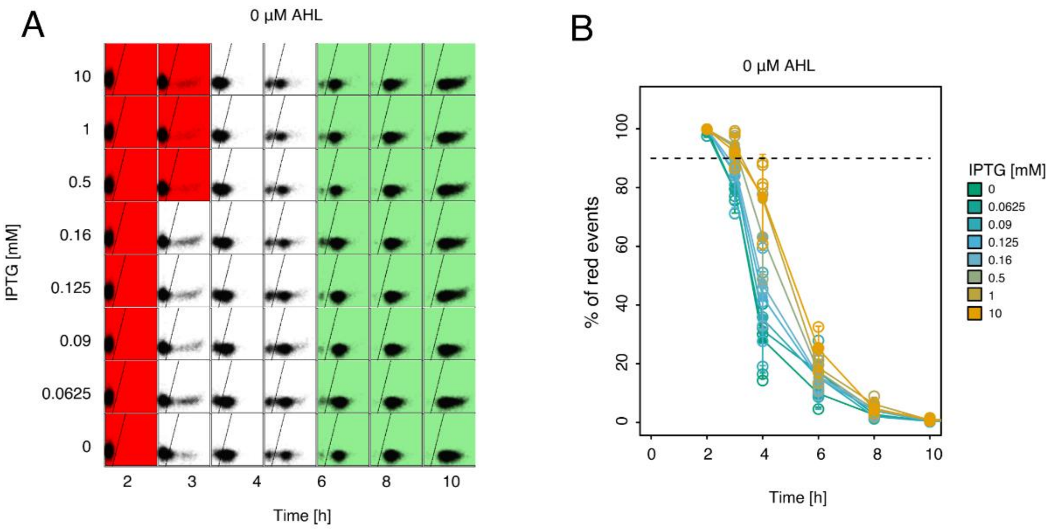

Appendix Figure S2: IPTG effect on the switching time of the red to the green state
A. Effects of IPTG concentration on the switching time from the red to the green state. Each square represents flow cytometer data of 10,000 events red (Y-axis) and green fluorescence (X-axis). The background color of the square indicates whether >90% of the events are in the red or the green gate.
B. Percentages of cells in the red gate over time. Mean (full circles) and standard deviation of 3 biological replicates (empty circles). The dotted lines represent the 90% threshold for the red color.
