# Supplementary material for: Controlling spatiotemporal pattern formation in a concentration gradient with a synthetic toggle switch
Source: Mol Syst Biol. 2020 Jun 12;16(6):e9361. doi: 10.15252/msb.20199361 (PMC7290156; doi:10.15252/msb.20199361)
Supplement: Supplementary file 2 — Expanded View Figures PDF [file MSB-16-e9361-s002.pdf]

Expanded View Figures

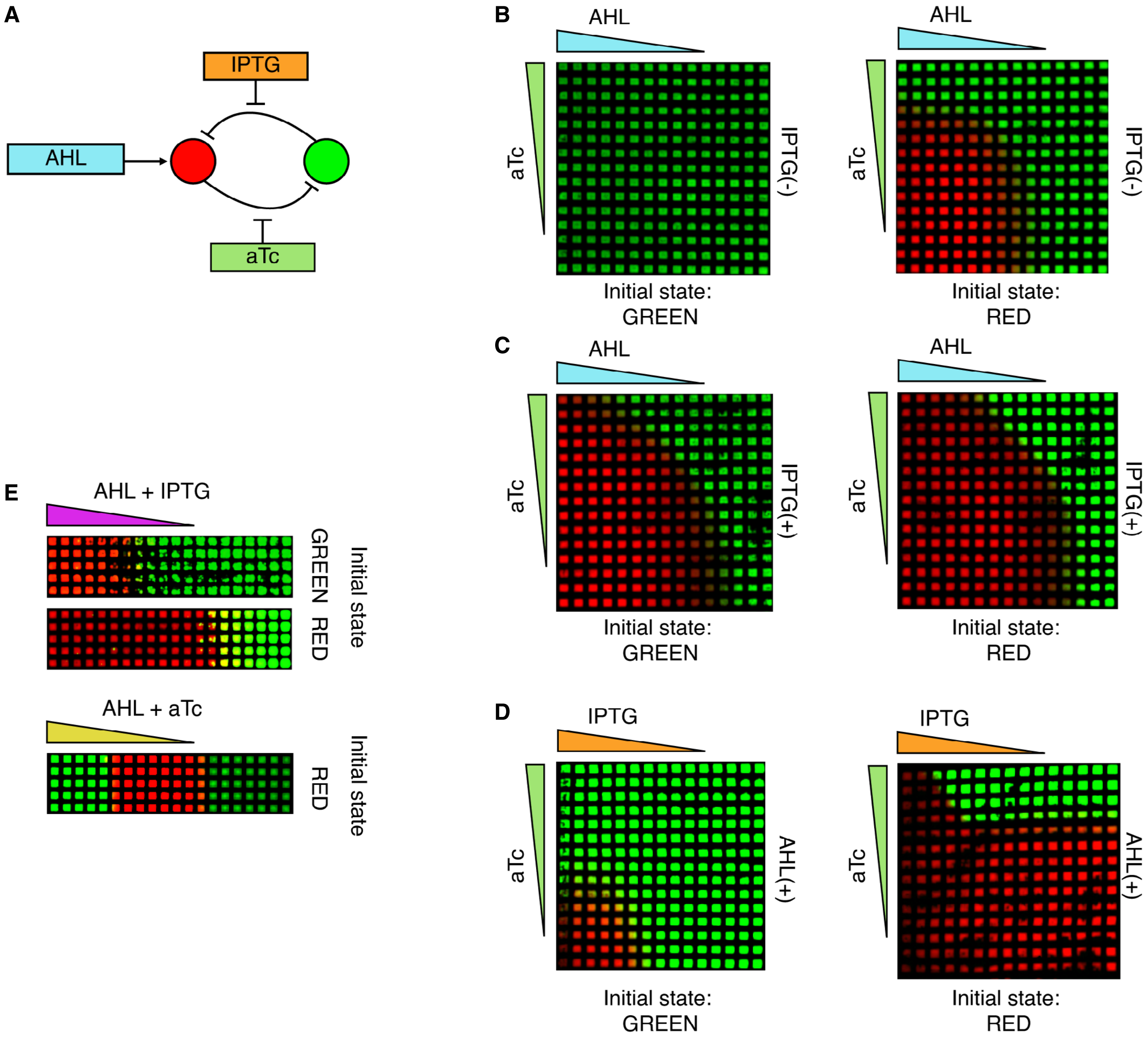

Figure EV1. Exploration of different patterns using the inducible toggle switch.

A Topology of the toggle switch network as described in Fig 1A with the addition of aTc that regulates the repression strength of the red node.

B, C Grid pattern of the circuit with the addition of 5 μl of aTc (5 ng/ml) and AHL (100 μM) at the indicated grid edges in the absence (B, 0 mM) or presence of IPTG (C, 10 mM) in the agar plate.

D Grid pattern of the circuit with the addition of 5 μl of 100 mM IPTG and 5 μl of 5 ng/ml aTc at the indicated grid edges in the presence of 10 μM of AHL in the agar plate.

E Patterning with parallel gradients. The inducer and the regulator were added together at the same edge of the grid, thus diffusing in parallel. This is equivalent to explore the pattern formed in the diagonal of the grids of Fig 1E and panel B.

The triangles outside the grid in B-E indicate the direction of the diffusion of IPTG (orange), AHL (cyan), aTc (green), AHL + IPTG (purple), and AHL + aTc (yellow). The colors correspond to the levels of fluorescence of mCherry (red) or GFP (green) produced by bacteria grown on a grid.

© 2020 The Authors

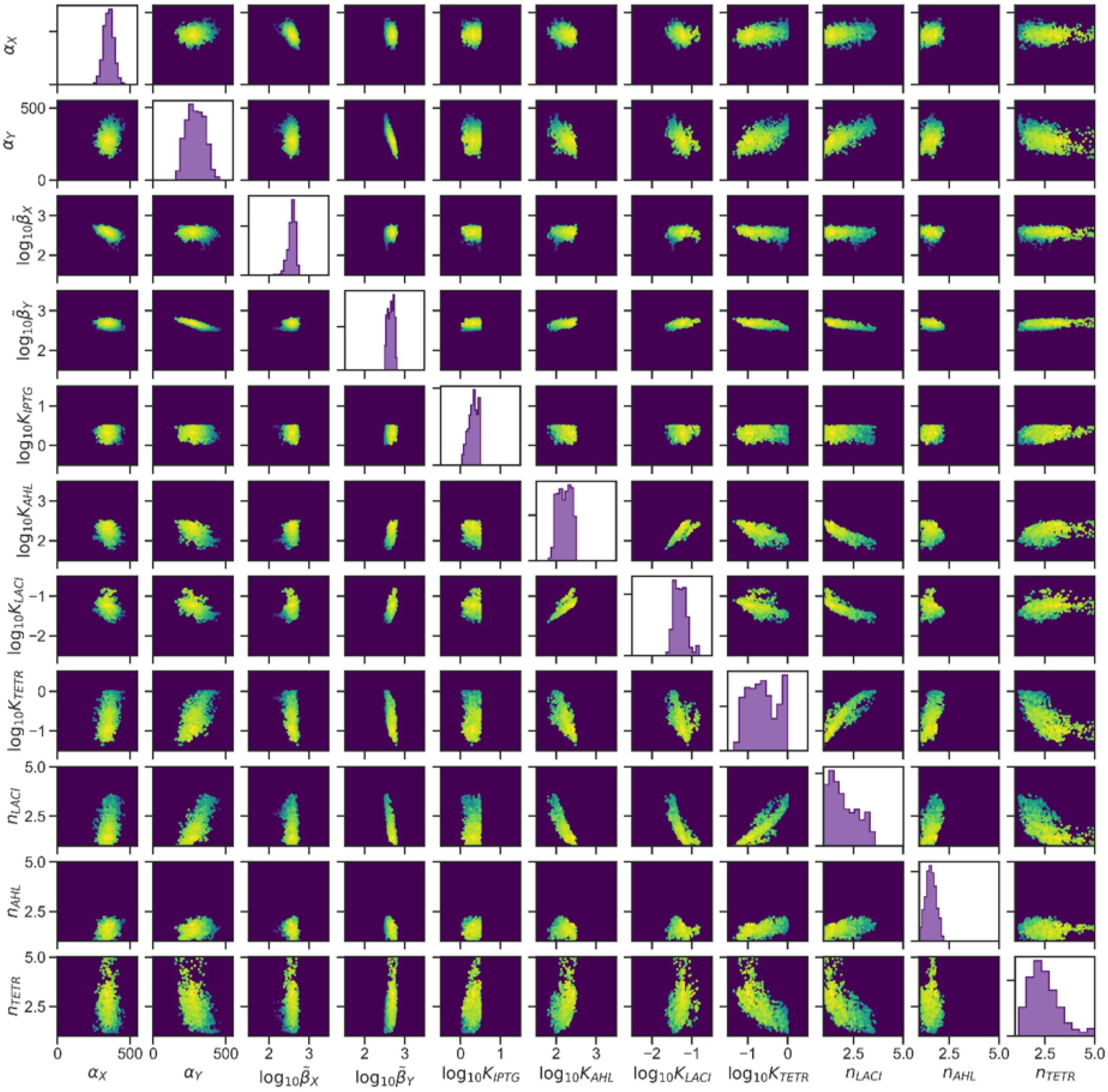

Figure EV2. Probability space of parameters.

Result from the MCMC fitting to the flow cytometry data. Diagonal shows posterior marginal probability distributions, and off-diagonal shows scatter plots from the successful points of the sampling of the posterior distribution, where color indicates likelihood magnitude.

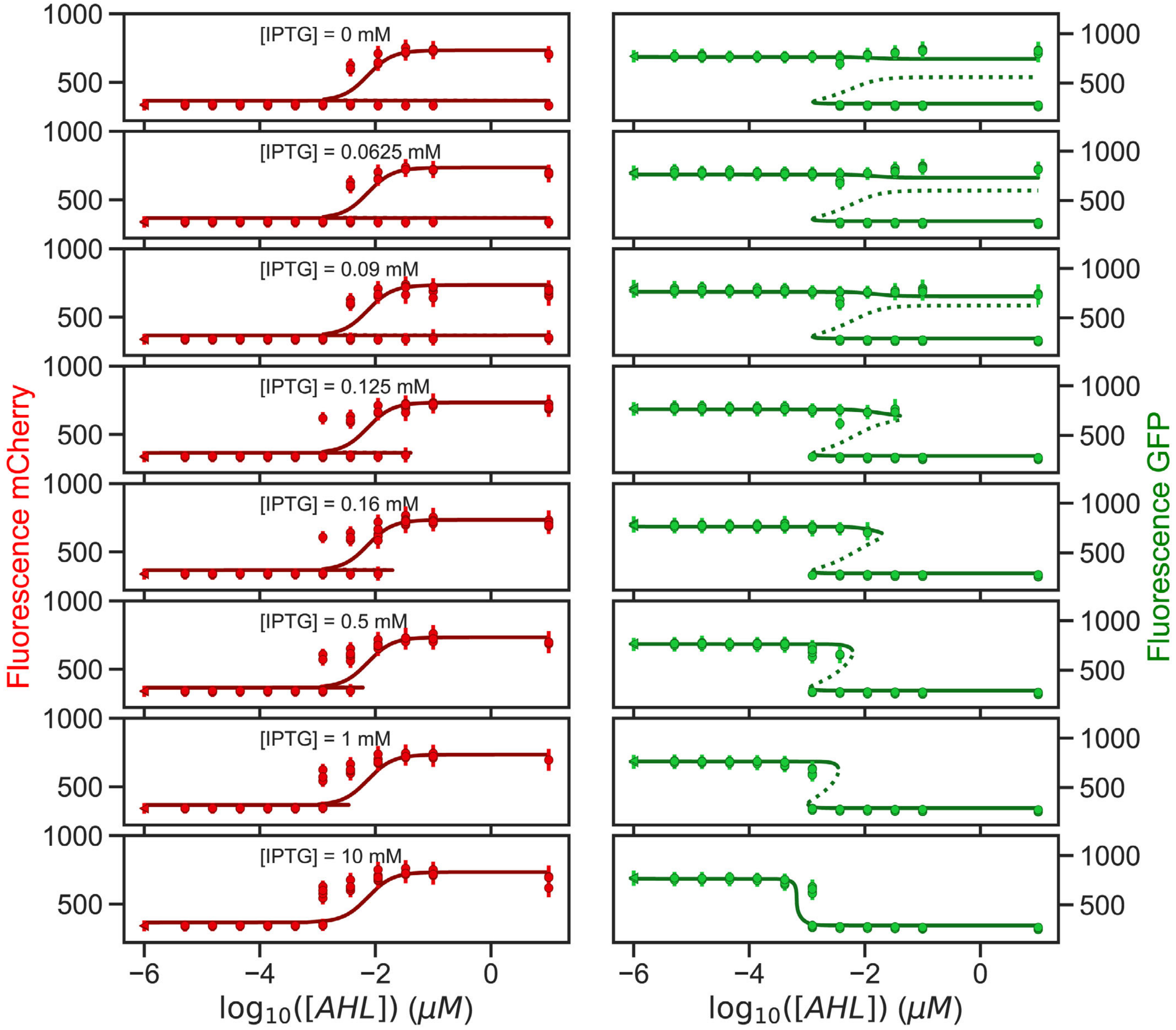

Figure EV3. Parameterization of the mathematical model.

Complete dataset of Fig 2B. Comparison between the observed populated states from the whole flow cytometry dataset (circles) and the available steady states predicted by the model (solid lines: stable states; dotted lines: unstable states). Experimentally observed states show the median and standard deviation for the different gated populations from three replicates. Parameters used in the model are the best parameter candidates from the MCMC fitting.

© 2020 The Authors

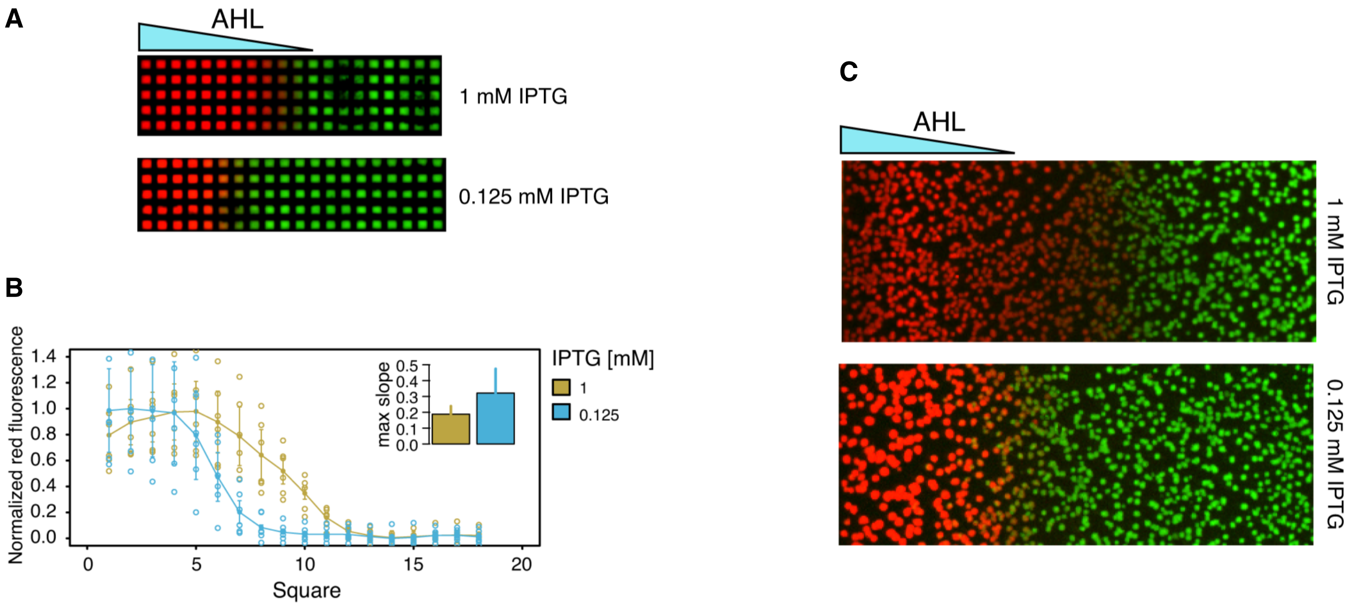

Figure EV4. Boundary precision when bacteria are growing on a solid surface.

A Grid assay at two different homogenous concentrations of IPTG, 1 mM for the sigmoidal regime and 0.125 mM for the bistable regime. Five microliter of a solution of 100 µM AHL was added at the left.

B Quantification of red intensity of six biological replicates, of which one representative replicate is shown in A. Shown are the mean (full circles) and standard deviation (error bars) of six biological replicates (individually shown as empty circles). The inset bar plot shows the mean of the maximum slope measured for each IPTG concentration from six replicates with standard deviation as error bars.

C Pictures of bacterial colonies harboring the inducible TS in the presence of homogenous IPTG concentrations (top: 1 mM; bottom: 0.125 mM) and in a gradient of AHL. To generate the gradient, 10 µl of 100 µM AHL was added on a paper disk placed on the left edge (not shown). Pictures were taken after overnight incubation (~ 16 h) at 37°C.

© 2020 The Authors

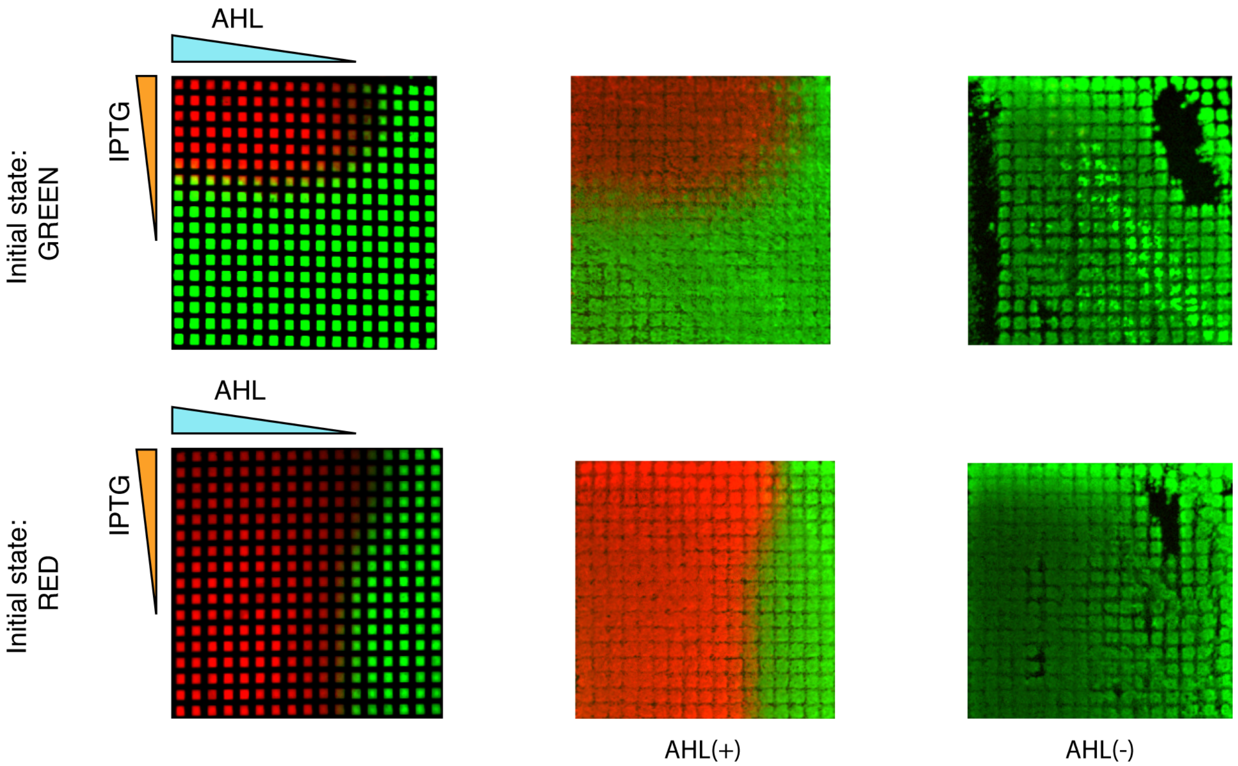

Figure EV5. Memory propriety of the toggle switch.

Demonstration of the memory property of the inducible toggle switch. Left: grid patterning reproduced as described in Fig 1. Middle: The cells of the left grid were transferred with a paper stamp onto a new grid placed on top of an agar plate supplemented with 5 μM of AHL. We observed that the pattern was maintained. Right: The cells of the left grid were transferred with a paper stamp onto a new grid placed on top of an agar plate without any IPTG or AHL. The pattern was lost (control). All images were recorded after overnight incubation (~ 16 h) at 37°C, triangles indicate the diffusion direction of the added molecules, and colors represent the presence of GFP (green) and mCherry (red).

© 2020 The Authors
